# Case of Induration of the Vesiculæ Seminales, Prostate Glands, and Rectum

**Published:** 1800-12

**Authors:** Caleb Crowther

**Affiliations:** Wakefield


### *To the Editors of the Medical and Phyſical Journal.*

GENTLEMEN,

IF the incloſed medical hiſtories and reflections correſpond with the plan of your Journal, the diſpoſal of them is very much at your ſervice. I am,

GENTLEMEN,

Your humble ſervant,

CALEB CROWTHER, M. D.

*Wakefield,*

*October* 18, 1800.

---

### *Caſe of Induration of the Veſiculæ Seminales, Proſtate Glands, and Rectum.*

I firſt ſaw Mr. A. B. a married man, aged about fifty, on the 19th of Auguſt, 1798. He was then affected with frequent motions to make water, accompanied with exquiſitely painful emiſſions of ſemen every time he made water; he complained alſo of frequent pain in the glans penis. His bowels were regular, he had no thirſt, his pulſe was about 80 in a minute, his appetite not much impaired; in ſhort, he followed his uſual occupation, and the only inconvenience he ſuffered was from the debility and emaciation induced by the painful emiſſions. The ſurgeon who had previouſly attended him, Mr. Taylor of this place, informed me that about the middle of February laſt, he began to be affected with ſtrangury, which continued with occaſional intervals of eaſe, until the latter end of April, when he was attacked with ſwelled teſticle, accompanied with conſiderable confiderable pyrexia. On the difappearance of this affection, came on the painful and involuntary emiffion of femen after voiding urine. After this fymptom had continued fome time, the femen emitted was nothing more than a light froth, followed by a difcharge of air from the urethra.

From this period, the 19th of Auguft, I faw him no more until the 25th of December, four days before his death. I was informed by Mr. T. that he continued in much the fame ftate as when I laft faw him, until about the middle of November, when the emiffion of femen ceafed. On the 1ft of December, he began to have much more pain than ufual about the neck of the bladder, his ftrength funk faft, his general appearance altered very much, and in the courfe of a few days afterwards he began to difcharge purulent matter from the urethra, which continued until his death.

### Appearances on Dissection.

On infpecting the cavity of the pelvis, the bladder appeared thickened, and adhered firmly to the rectum and adjacent parts. The upper part of the rectum was thickened and indurated, or, as it is commonly termed, fchirrous. The veficulæ feminales were about the fize of a moderate nutmeg; when cut into, there appeared no regular cavity, but extravafated blood feemed to be interfperfed through their whole fubftance.

Both the proftate glands were much enlarged and indurated, and the left gland in a ftate of fuppuration. The urethra was found throughout the whole courfe, but confiderably contracted near the neck of the bladder. The internal furface of the bladder was not ulcerated nor difeafed. The tefticles were both found, but very fmall. The epididymis of one of the tefticles appeared to be wafted away, in the other tefticle it was very fmall.

The only remedy which I ordered for him was cicuta and opium, in the form of pills, which he took but for a very fhort time. He afterwards took cicuta and calomel; and a neighbouring phyfician, who confidered the cafe to be an ulceration of the bladder, ordered for him uva urfi, mucilaginous medicines, and anodyne injections. The only thing which afforded him relief was opium. In the early ftage of the difeafe he took various other medicines appropriated to the exifting fymptoms of the complaint.

He confeffed that he had been much addicted to Onanifm in his youth, and warned his young relatives a few days before his death of the pernicioufnefs of the practice, to which he afcribed his prefent complaints. It is not improbable, I think, that his furmife with refpect to the caufe of his difeafe was well well founded. On examination per anum in Auguft, I could not difcover any enlargement in the proftate glands. The diminution in the fize of the tefticles was effected by a continuation of the difeafe; there was nothing unnatural in their appearance when I firft faw him. The difeafed part of the rectum was not contracted; but if not kept open in his bowels, he had frequent tenefmus.

Suppofing that a cafe precifely fimilar to this were to recur, and that the medical attendant fhould be thoroughly acquainted with the parts difeafed, the profpect of a cure from the exhibition of medicines would not, I fear, be very favourable.

